# Cardiogenic Shock Due to Atrial Myxoma With Mitral Valve Involvement

**DOI:** 10.7759/cureus.28520

**Published:** 2022-08-29

**Authors:** Michael Maiden, Joshua Payne, Anna Shu

**Affiliations:** 1 General Surgery, St. Joseph Mercy Oakland Hospital, Pontiac, USA

**Keywords:** surgery, atrium, cardiogenic, mitral, shock, myxoma

## Abstract

Despite being the most common primary cardiac neoplasm, the incidence of cardiac myxomas remains low. The majority of myxomas usually have a nonspecific presentation often leading to symptoms such as cough, dyspnea, and weakness. Larger tumors may cause arrhythmia, syncope, or cerebrovascular events due to embolization. Rarely, patients with myxomas may present with signs and symptoms of cardiogenic shock. A 50-year-old female presented to our Emergency Department with an altered mental status and hypotension. Initial imaging of the patient’s head showed an embolic infarction. Subsequent investigations revealed a large atrial mass protruding through the mitral valve. The patient was initially resuscitated and then taken to the operating room emergently where the mass was removed. Postoperatively, she was observed in the intensive care unit and eventually transferred to a step-down unit. Her pathology report confirmed that the mass was a cardiac myxoma. Here, we report the case of a patient with an atrial myxoma protruding through the mitral valve who presented in cardiogenic shock. The etiologies of cardiogenic shock and atrial myxomas are explored. The medical and surgical management of a patient with an atrial myxoma presenting in cardiogenic shock is reviewed. We reflect on our diagnostic evaluation, determining that patients who present in undifferentiated cardiogenic shock should be approached with a broad differential diagnosis.

## Introduction

Cardiac myxomas are the most common primary cardiac neoplasm. However, the incidence and prevalence of myxomas are estimated to be low. Based on autopsy studies, the estimated incidence of myxomas is between 8 and 150 per million [1]. In a retrospective review, patients with myxomas most commonly presented between the third and sixth decades of life, and were significantly more common in females [2]. Clinical manifestations of cardiac tumors are determined by their size, location, and mobility [3]. Approximately 75% of cardiac myxomas are located in the left atrium, 23% in the right atrium, and 2% within the ventricles [4].

The most commonly reported symptom of myxomas is dyspnea [5]. Other frequently reported symptoms include cough, paroxysmal nocturnal dyspnea, hemoptysis, and constitutional symptoms (i.e., fever, weight loss, etc.) [6]. Auscultation abnormalities are present in the majority of patients, with the most common being a characteristic “tumor plop” heard in diastole [5,6]. Left atrial enlargement is frequently seen on electrocardiography while cardiomegaly is commonly seen radiographically [5].

As previously discussed, the most common location for a cardiac myxoma is the left atrium, typically in the zone of the fossa ovalis [6]. The clinical presentation of cardiac myxomas located in the left atrium can vary from an asymptomatic incidental mass to signs of obstruction of the mitral valve with right heart failure [6]. Symptoms such as dizziness and syncope are related to intermittent obstruction of the mitral valve by the mass. Larger myxomas extending through the mitral valve causing complete obstruction can result in sudden cardiac arrest, death, or myocardial infarction from coronary artery emboli [7].

Cardiogenic shock is a consequence of cardiac pump failure, resulting in decreased cardiac output and increased systemic vascular resistance. There are numerous causes of cardiac pump failure which can be broadly divided into four categories, namely, myopathic, arrhythmic, mechanical, and extracardiac [8]. Some studies have estimated that over 70% of cases of cardiogenic shock are due to myocardial infarction [9]. Other commonly examined causes of cardiogenic shock are decompensated heart failure, pulmonary emboli, drug intoxication, myocarditis, and endocarditis [8,9]. There are few published case reports of atrial myxomas causing mechanical cardiogenic shock. This article contributes to the literature by discussing a rare presentation of cardiac myxomas while reviewing the surgical management of cardiac myxomas and exploring the etiology of myxomas and cardiogenic shock.

## Case presentation

A 50-year-old female presented to our Emergency Department with chest pain and paroxysmal nocturnal dyspnea. Her only pertinent history was heavy substance and alcohol abuse reported by her accompanying family. She was disoriented upon presentation, oscillating between an obtunded state and active hallucinations. Initially tachycardic and hypotensive, she quickly required multiple vasopressors to maintain her mean airway pressure (MAP) goals. Laboratory investigations revealed a profound lactic acidosis and elevated troponin T. Due to her acidotic state, hemodynamic instability, and altered mental status, the patient was intubated for airway protection.

**Figure 1:**
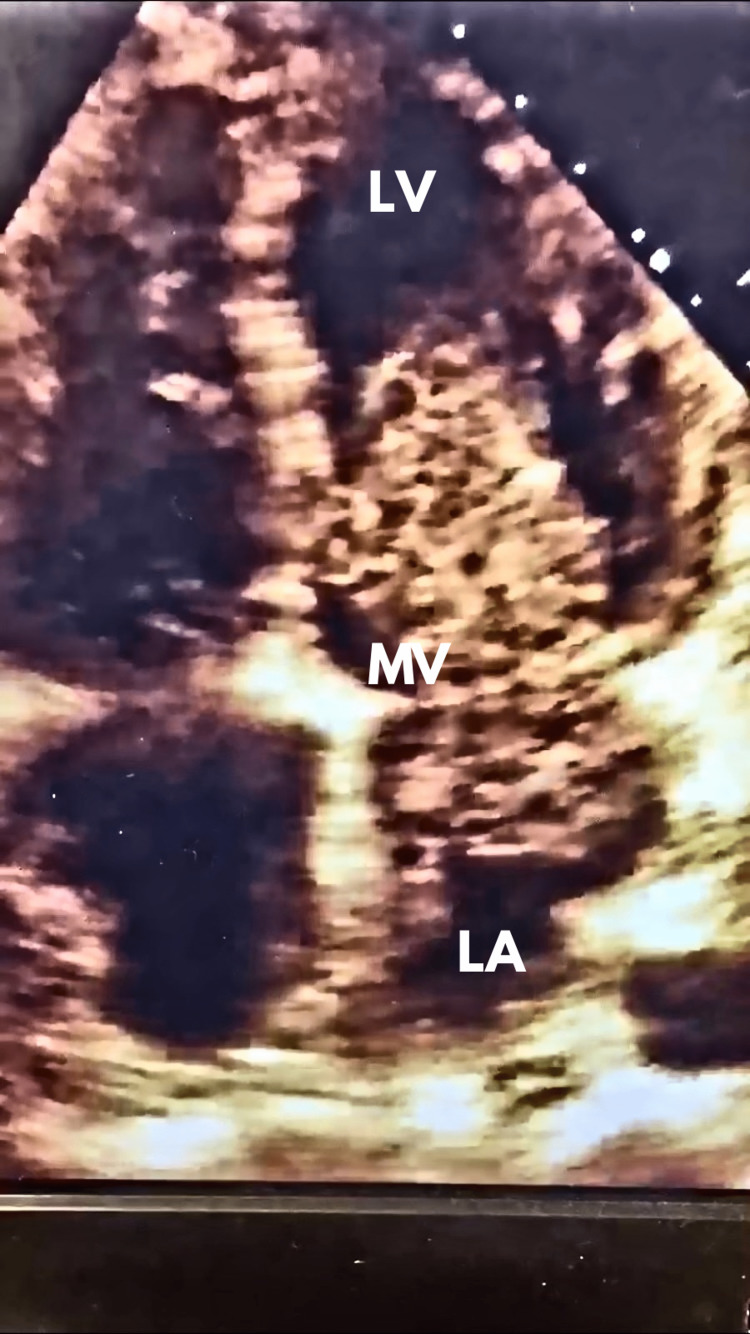
Photograph of TTE demonstrating myxoma protruding through the patient’s mitral valve. LA: left atrium; LV: left ventricle: MV: mitral valve; TTE: transthoracic echocardiogram

Initial imaging with chest radiography and abdominal computerized tomography (CT) uncovered a left lung infiltrate and small bilateral pleural effusions. A 12-lead electrocardiography showed sinus tachycardia with ST depression in leads V3-V5. CT imaging of her head unveiled a likely embolic infarction. A transthoracic echocardiogram (TTE) revealed an extremely large mass in the left atrium protruding through her mitral valve (Figure 1).

**Figure 2:**
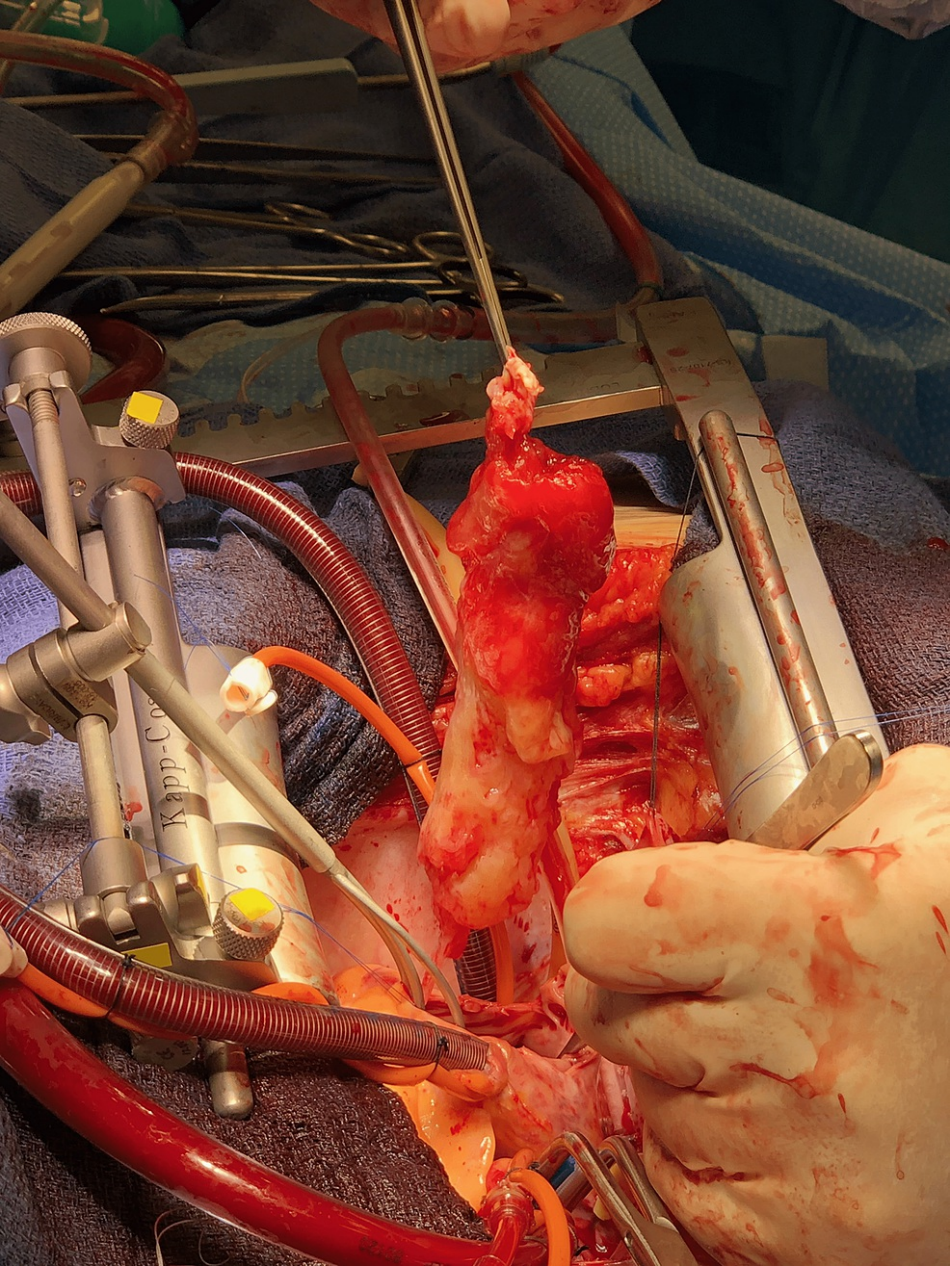
Surgical specimen extracted during the procedure. Pathology later confirmed the mass to be a benign cardiac myxoma.

She was taken to the operating room emergently where her aorta was cannulated. She was placed on cardiopulmonary bypass and oxygenated for 10 minutes prior to the aorta being clamped. Cardioplegia was administered in an antegrade fashion. A median sternotomy was performed. The pericardium was opened, and an incision was made through the right atrium. The atrial septum was opened and the masswas removed without damaging the mitral valve (Figure 2).

A large atrial septal defect remained after the stalk was removed and repaired with a Dacron patch. She received four units of packed red blood cells, four units of fresh frozen plasma, one unit of platelets, and one unit of cryoprecipitate. After spending two days in the surgical intensive care unit, the patient was transferred to a cardiac step-down unit. She recovered well but was significantly debilitated from her surgery. She was transferred to inpatient rehabilitation and eventually discharged. Pathology confirmed that the mass was a cardiac myxoma measuring 10.5 × 4 × 2.5 cm with no evidence of metastatic disease.

## Discussion

The diagnosis of a cardiac myxoma is challenging given the diverse and variable clinical presentation. In a retrospective observational study at a single center over a decade, palpitations were the most commonly reported symptom whereas 74% had a mid-diastolic murmur [10]. Stroke is another very common presentation due to thromboembolic events. No patients presented with cardiogenic shock, and few presented with signs and symptoms of heart failure.

Cardiogenic shock is characterized by end-organ hypoperfusion as a consequence of inadequate cardiac output. Quantitative parameters used to define cardiogenic shock vary, but usually include systolic blood pressure of ≤90 mmHg, or requiring vasopressor support to maintain adequate perfusion and evidence of end-organ hypoperfusion [11]. Cardiogenic shock continues to have an extremely high mortality rate, ranging from 40% to 72% [12]. Diagnostic and therapeutic interventions are typically determined by the severity of shock [13]. However, certain therapies are dependent on the etiology and diagnostic efforts should be targeted at characterizing cardiac dysfunction [14]. Echocardiogram and cardiac catheterization are the preferred diagnostic modalities as they provide extensive information regarding cardiac function [14]. Various treatments exist and as stated prior depend on the severity of shock and etiology of the cardiac insult. Treatment may include medical therapy (inotropes and vasopressors) but may require invasive interventions such as intra-aortic balloon pumps or extracorporeal membrane oxygenation.

Echocardiogram is a key diagnostic modality when attempting to determine cardiac dysfunction and is essential in the diagnosis of a myxoma. TTE has a reported sensitivity of 95% with a near 100% sensitivity for cardiac myxomas [15]. Echocardiography can also characterize the size, location, mobility of the mass, and the extent of obstruction of circulation. Positron emission tomography scans and cardiac CT/MRI are alternative diagnostic modalities. Coronary angiography is imperative and can provide valuable information regarding the tumor’s blood supply [15]. If surgical resection is planned, angiography is an essential part of the preoperative evaluation. During the initial evaluation of our patient, our clinical efforts were aimed at resuscitation. Once evidence of an embolic infarct was relayed to the surgical team, a TTE was ordered immediately. Maintaining a broad differential diagnosis when encountering patients in undifferentiated shock allows the provider to evaluate all potential causes of shock and minimize delays in diagnosis.

Surgical resection is the treatment of choice and is usually curative [16]. In a single-center retrospective review, 74 patients underwent complete excision of their primary intracardiac myxoma over 30 years [16]. There were no intraoperative mortalities. The authors did report two early mortalities (due to cerebral coma and hepatorenal syndrome) and 12 late mortalities. There were no recurrences. Access to the heart varied given tumor characteristics but the surgical technique was standardized and included median sternotomy, total cardiopulmonary bypass, hypothermia, and cardiac arrest using crystalloid cardioplegia [16]. Patients are routinely followed up on an outpatient basis. Follow-up typically includes clinical examination, electrocardiography, and echocardiography.

## Conclusions

Cardiogenic shock caused by a cardiac myxoma is an uncommon presentation of a cardiac tumor and a rare etiology of cardiogenic shock. Cardiac myxomas are difficult to diagnose due to their varying clinical presentation. Echocardiography is highly sensitive and specific for cardiac myxomas and may aid in diagnosis. Surgical resection is the treatment of choice.
